# Analysis of the Effects of Tandem Welding (Fronius TPS/i - TWIN) of S1100QL and S1300QL Steels

**DOI:** 10.3390/ma18112577

**Published:** 2025-05-31

**Authors:** Mateusz Karczewski, Krzysztof Mroczka, Sławomir Parzych, Piotr Bała, Grzegorz Cios, Janusz Mikuła, Grzegorz Jeż

**Affiliations:** 1 Faculty of Materials Engineering and Physics, CUT Doctoral School, Cracow University of Technology, Warszawska 24 Str., 31-155 Kraków, Poland; mateusz.karczewski@doktorant.pk.edu.pl (M.K.); grzegorz.jez@doktorant.pk.edu.pl (G.J.); 2 Faculty of Materials Engineering and Physics, Cracow University of Technology, Warszawska 24 Str., 31-155 Kraków, Poland; slawomir.parzych@pk.edu.pl (S.P.); janusz.mikula@pk.edu.pl (J.M.); 3 Faculty of Metals Engineering and Industrial Computer Science, AGH University of Krakow, Mickiewicza 30 Av., 30-059 Kraków, Poland; pbala@agh.edu.pl; 4 Academic Centre for Materials and Nanotechnology, AGH University of Krakow, Mickiewicza 30 Av., 30-059 Kraków, Poland

**Keywords:** tandem welding, Fronius TWIN, high-strength steels, martensite and bainite microstructure

## Abstract

S1100QL and S1300QL steels are classified as fine-grained steels with a low-carbon martensitic structure. Tandem welding is a method of creating a joint by melting two electrode wires in a one-behind-the-other configuration. This article presents the effects of creating dissimilar joints, elements of varying thicknesses made from S1100QL and S1300QL steels. The analysis focused on temperature changes in the heat-affected zone (HAZ) during welding, as well as the macro and microstructure, and the properties of the joints created at welding speeds of 80, 90, and 100 cm/min. The shortest cooling time (t8/5) in the HAZ for S1300QL steel was 9.4 s, while the longest was 12.4 s. Thermal cycle simulations were performed for the analyzed materials, with a cooling time of 5 s. The test results demonstrated that TWIN welding was stable, and an optimum welding speed is 80 cm/min. The HAZ microstructure for the highest cooling speed (t8/5 = 5 s) of S1100QL steel contains, in addition to martensite, lower bainite, while S1300QL steel consists of martensite. Tempered martensite was also detected at slower cooling rates. For all speed variants, the impact energy is above 27 J at a test temperature of −40 °C. In turn, hardness tests showed that the base material for both steels has the highest hardness. However, the lowest hardness was found for the weld.

## 1. Introduction

Microstructure The main objective of welding ultra-high-strength steels is to introduce small changes in their microstructure due to the thermal cycles of welding. This concerns the heat-affected zone (HAZ) of the welded joint. The strength of the material depends on the strengthening mechanisms—grain refinement (grain boundaries), dislocation, precipitation, and solution. For steels from S420 and with a higher yield strength, an acicular microstructure occurs. This is tempered martensite or martensite [1]. The finer this microstructure (the size of the so-called grain) is, the higher the strength. This mechanism is often supported by increased dislocation density, which occurs especially in martensite [2,3]. With increased dislocation density and thus facilitating diffusion, strengthening precipitates are more easily formed [4], but at the same time, dislocations can be a trap for hydrogen, limiting its diffusion [5]. This mechanism, i.e., precipitation strengthening, is related to the presence of Nb, V, and Ti in S1100QL and S1300QL steels [6,7]. In the case of the solution strengthening mechanism, each alloying element, the atoms of which (at least some of them) do not enter the composition of the precipitate, leads to solution strengthening. In S1100QL and S1300QL steels, these are mainly C, Mn, Si, Ni, Cr, V, and Mo, the contents of which in these steels are mostly significant. It should be noted that the mentioned steels are steels with a chemical composition rich in alloying elements such as Ni, Mo, or V, but also steels in which the primary alloying element is Mn [8,9,10,11,12].

Weldability The limitation for increasing alloying additions in the case of structural steels (S) is that they are sufficiently well weldable. In the case where chemical composition is taken into account, the weldability is determined by the carbon equivalent (CEV). According to [13] and MIS (International Welding Institute), it is calculated according to the following equation [9]:

(1) $$\mathrm{CEV}=C+\frac{\mathrm{Mn}}{6}+\frac{\mathrm{Cr}+\mathrm{Mo}+V}{5}+\frac{\mathrm{Ni}+\mathrm{Cu}}{15}\%\mathrm{wt}$$

The lower the CEV calculation result, the better the weldability of a given steel, but a result above 0.45% indicates poor weldability. It is worth recalling here that the concept of weldability is much broader than just an assessment based on chemical composition. It has been noted that obtaining a welded joint that meets expectations depends on three factors: metallurgical, structural, and welding technology. The first of these includes chemical composition and microstructure, including phase transformations and imperfections. The second refers to parameters, e.g., welding method, linear energy, or pre-welding heating. The third concern is weld length, joint shape, stiffness conditions of the structure, and thickness of welded elements. The definition that combines these three aspects was developed at the Institute of Welding Gliwice (currently SBŁ GIT Centre of Welding): Weldability is the ability to create joints by fusion welding with the required physical properties, capable of carrying the loads intended for a given type of structure for which the given steel is to be used [14]. For this reason, especially with regard to steels such as S1100QL and S1300QL, for which the CEV is often above 0.4% [9], various welding methods and conditions are sought to enable welding of these steels.

Welding Maintaining the microstructural features described above under welding conditions is difficult due to the need for a temperature above the melting point at the joint. Therefore, various solutions are sought that enable the joining of steel elements with the smallest possible amounts of energy introduced to the material. A promising technology is friction stir welding (FSW), which was initially developed for joining light alloys such as aluminum or magnesium [15,16,17,18,19]. However, the development of new materials for the FSW tools already enables the welding of various types of steel [20,21,22,23], including high-strength steels [24]. The advantage of FSW technology is the welding of flat elements (similar to fusion welding), but the process is carried out in the solid state, which means that it does not require partial melting of the joined materials. FSW is performed by the rotation of the FSW tool, which moves (linearly) along the edges of the joined elements. This causes the joined materials to be mixed and metallurgically joined. The basic phenomenon of this process is plastic deformation. Due to some limitations of FSW, fusion welding is still the dominant technology for welding steel. Among these methods, Gas Metal Arc Welding (GMAW) is dominant. The GMAW method is based on melting the joined materials by an electric arc that glows between the joined materials and the electrode. The electrode is also a filler, because it melts during welding. The liquid metal of the weld pool is protected by a shielding gas that flows out of the nozzle, from which the electrode wire is also automatically ejected. The GMAW is related to the possibility of making welds manually but also fully automatically, e.g., in robotic systems [25]. It is also an efficient method, especially when the weld consists of a single bead. However, when it is necessary to join materials with a thickness of more than 3–4 mm, it is necessary to specially prepare the edges of the joined elements (e.g., V, Y, or X chamfering) and to make a larger number of passes. This causes multiple liquefactions and associated multiple thermal cycles. The answer to these challenges is, for example, hybrid systems, which combine laser welding with GMAW [26]. Laser radiation pre-melts the edges of the materials being joined. This causes the GMAW process to reach much deeper. Such a system allows for the preparation of the edges of the joined elements to I (without chamfering) and the production of a joint with full penetration of thicker elements, but also with the shape of the face, as in the case of conventional single-pass welding. Complete penetration of the welded materials in a narrow thermal range but with steep temperature gradients is also achieved using only laser action [27,28]. These solutions are interesting but require an extensive station equipped with a welding laser. When discussing new solutions in welding, the use of artificial intelligence (AI) is also worth mentioning. In article [29], the authors highlight the use of this technology to predict welding outcomes. Due to the rapid development and widespread adoption of various AI-based solutions in recent years, it is worth paying attention to this technology.

TWIN Another interesting solution in welding technology is engaging more than one welding wire in one welding process. Such solutions in various configurations are used in submerged arc welding. The concept of using two welding wires (electrodes), separately powered and controlled by current in one welding process, has been transferred to GMAW welding [30,31]. The method is called TWIN-wire or tandem welding. The welding torch is designed to feed the wires towards the weld pool at a set angle to each other—0, 4, 8, or 11 degrees. The weld pool is protected by a shielding gas, the flow of which is about 60–80% greater than in the case of single-wire welding. The torch moves relative to the weld groove so that the wires weld one behind the other [32]. For this reason, the first wire (relative to the welding direction, called Lead) usually performs the penetration and preheats the welded materials. Hence, welding with this wire is usually carried out with the use of higher energy. The second wire (called Trail) is to fill the weld groove, and therefore, the amount of energy supplied to this wire is lower. See Section 3.

It should be noted that the welding variant of parallel wire guidance relative to the welding direction is also investigated [33].

The advantage of the TWIN method is primarily the improvement of welding efficiency, which consists of increasing penetration, welding speed, and the amount of deposited metal. Other advantages include: reducing the amount of heat input, better heat distribution during welding, reducing the number of thermal cycles in the case of welding thick profiles, reducing microstructural defects due to contamination of the bead surface before the next bead is laid, better regulation of current flow, stabilization of arcs by induction caused by different currents flowing through each of the wires, creation of favorable conditions for the separation of liquid metal drops as a result of the presence of the second arc [32,34,35,36]. Disadvantages include the possibility of adverse impact of electric arcs on each other by their repulsion and generation of instability, which can lead to spatter and uneven melting of electrodes [34]. Due to steeper thermal cycles (faster cooling), the impact strength in the heat-affected zone (HAZ) of some steels is lower compared to that obtained after conventional welding [32]. The disadvantages also include the need to use two power sources and automated systems, e.g., a welding robot [33], although there are already reports of a manually operated TWIN-wire welding machine. The general advantage of the TWIN-wire system is better efficiency and better quality of joints, although the method has its limitations.

CMT technology was developed in response to the need to reduce the amount of heat introduced into the material and to increase control over the transfer of molten wire electrode material to the weld pool. The method is a variation in short-circuit transfer. The essential feature of CMT is the reciprocating movement of the wire with a frequency between 120 Hz and 170 Hz, which depends mainly on the synergistic line. In the first years of introducing CMT technology, the frequency was lower—70 Hz [37]. Such action promotes the detachment of the droplet from the wire and its transfer to the weld pool [38]. This happens when the droplet contacts the welded material with the wire, when the electric arc is extinguished. At this time, the voltage drops to zero, and a small short-circuit current flows. Periodic extinction of the arc significantly reduces the amount of heat generated during welding, which is emphasized in the name of the CMT process—cold metal transfer. Regardless of the movement of the wire, it is also important to control the arc while it is glowing. Taking this into account, the following are distinguished: CMT-MIX (CMT-Pulse)—pulse control, CMT-AC (CMT-Advanced; otherwise known as variable polarity-VP)—change in polarity, CMT MIX + Synchropulse (CSP)—high pulse frequency at a higher average welding current, which increases the arc force and the energy of transferring the droplet of liquid metal [38,39,40,41,42,43]. The process has a number of advantages: minimal heat input to the material, limited amount of spatter, process stability and control, droplet transfer control, good weld quality, possibility of welding materials in a wide range of their thicknesses, high welding speed, possibility of welding, surfacing, brazing and 3D production, efficient use of material and energy [44,45,46,47,48,49,50]. The CMT method also has limitations, especially when the process parameters are not optimally selected. The main disadvantages include: limitations in welding very thick materials, the risk of process instability at higher wire feed speeds and currents, and difficulty in selecting parameters and, for some processes, their low universality, i.e., correction when the temperature of the welded material increases [39,51,52,53,54].

Objective The research results described in this article are part of a broader study on developing a technology for manufacturing telescopes for mobile cranes, i.e., those mounted on truck chassis. In these solutions, the crane (hoist) has the form of a telescope to easily change the configuration of the machine from working to transport. The technology was developed using the Fronius TPS/i inverter welding machines, which form the basis of the tandem welding system [55]. The TWIN system has two types of welding machines: the Fronius TSP 500/i and TPS 600/i. The use of the TPS 600/i welding power source allows for welding with a current of 500 [A] at a 100% duty cycle of the device at a temperature of 40 °C, which is particularly significant in a robotic, high-performance welding process [56]. The Fronius TPS/i PUSH PULL CMT welding configuration was used for the welding experiments. Due to the different thicknesses of the materials being joined, a combination of PMC (Pulse Multi Control) PCS processes was chosen. At the specified current values, this process adopts the characteristics of spray transfer of molten metal in the electric arc and the standard PMC Universal process, which is a type of pulsed arc [57]. The base material is heat-treated steel S1100QL and S1300QL. When welding such materials, it is essential to maintain a high technological standard due to the potential formation of hardening structures in the joints, which significantly reduce the impact strength. The requirements set by modern welding industries aim to ensure high-quality joints while significantly increasing process efficiency. This is achieved by increasing welding speeds, reducing the number of passes, or eliminating thermal treatment processes.

To explain the influence of selected parameters and welding conditions on the applied steels, in addition to studies on welded joints, thermal cycle simulation experiments were conducted. The simulation parameters were determined based on the welding processes. In this way, a research material was obtained that contained one type of microstructure over a larger volume, which enabled more accurate studies of the microstructure and its mechanical properties. The main objective of this study was, therefore, to determine the microstructure and properties of S1100QL and S1300QL steels subjected to welding thermal cycles under conditions appropriate for the developed technology of manufacturing the crane telescope. Based on these studies, the optimal welding parameters were also selected.

## 2. Base and Filler Materials

The tests were conducted on WELDOX S1100QL and WELDOX S1300QL steels. The chemical composition of the steels is provided in Table 1 and Table 2, while the mechanical properties are presented in Table 3, where YP—yield point, UTS—ultimate tensile strength, KV—impact energy (test value energy), HV10—Vickers hardness 10 kg load.

**Figure 1:**
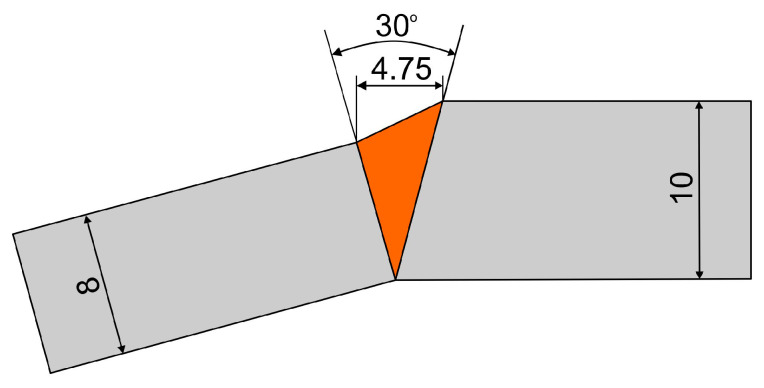
Welded joint—dimensions.

The material was in the form of plates with dimensions 1000 mm × 400 mm and a thickness of 10mm—S1100QL steel, and 1000 mm × 400 mm and a thickness of 8 mm—S1300QL steel. The welding was performed along the rolling direction in the configuration shown in Figure 1. The elements were prepared in a V shape with an opening angle of 30°. The components were joined together using tack welds. Root welding was performed using method 135 (MAG).

The filler material used was BÖHLER alform 700-IG, standard [58], AWS A5.28/SFA-5.28, G 79 5 M21 Mn4Ni1.5CrMo, ER110S-G, with a diameter of 1.0 mm. The chemical composition of the filler metal is presented in Table 4, and the mechanical properties are shown in Table 5.

## 3. Methodology of Welding Experiments and Research

**Figure 2:**
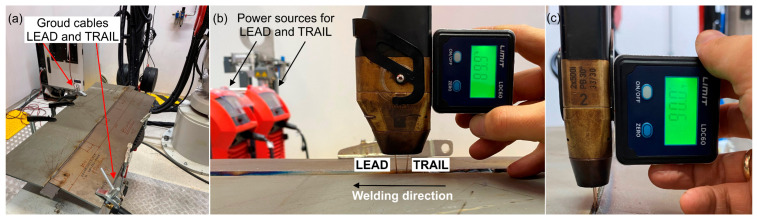
Welding experiment preparation—(**a**) welding station, (**b**,**c**) positioning the torch in the Y-axis and X-axis.

Welding was carried out on a robotic workstation equipped with an industrial welding robot from ABB and welding power sources from Fronius. In Figure 2a, the method of mounting the test plate on the surface of the positioner and the locations for connecting the ground cables are shown, power source no. 1 LEAD, power source no. 2 TRAIL. The setup of the torches is shown in Figure 2b,c. While the parameters used in the welding process are provided in Table 6.

The main welding was performed using the TWIN process, 72 h after the root pass welding was completed. During the experiment, the following sample designations were used—Table 7.

Visual tests (VT) were performed according to quality level B, as per standard [59]. Other documents related to the inspection include standards [60,61,62,63] Testing conditions: lighting intensity: 700 lx, observation distance: 650 mm, temperature: 20 °C. Instruments used during the tests: universal weld gauge, lux meter, caliper. No discrepancies were found after the tests.

Before performing the microscopic observations, the joint samples were ground and mechanically polished. Adler reagent and Nital reagent (4% HNO_3_ solution in methanol) have been used to reveal, respectively, macro-structure and microstructure. Macro and microscopic observations were made using a Keyence 7000-VHX digital light microscope (manufactured by Keyence Corporation Osaka, Japan). Documents related to the inspection include standards [63,64]. Fracture analysis was performed using a Jeol JSM-IT200 scanning microscope (manufactured by JEOL Ltd, Tokyo, Japan).

The SEM microstructure and EBSD analysis were carried out using Helios 5 PFIB CXe (manufactured by Thermo Fisher Scientific, Hillsboro, OR, USA) equipped with a Symmetry S3 EBSD detector (manufactured by Oxford Instruments Nanoanalysis, High Wycombe, UK). Data were acquired using an accelerating voltage of 20 keV, a spot size of 4, a step size of 100 nm, and a beam current of 48 nA in the “Speed 2” mode of the Symmetry S3 detector using a pattern resolution of 156 × 128 px. The registered patterns were background corrected, and typical Hough-based indexing was performed with up to 12 Kikuchi bands detected.

Hardness measurements were carried out using the Vickers-Brinell hardness tester, type HPO-250, in the Vickers configuration, under a load of indenter of 10 kg (HV10) in a time of 10–15 s. The acceptance criteria, according to the technical reference documents, are a hardness of the weld material and HAZ < 452/450 HV10. Documents related to the hardness testing include standards [63,65].

Tensile testing of the transverse samples was carried out on a testing machine ZD-40 (400 kN), in accordance with the standards [63,66]. The width of the parallel length of the tested samples was 25 mm.

The impact test was performed using a Charpy impact testing machine with a dial gauge, on samples with dimensions of 55 × 10 × 7.5 mm^3^ and a V-notch. The test temperature was −40 °C, with methanol and liquid nitrogen as the cooling agents. Documents related to the impact testing include standards [63,67].

The thermal cycle analysis was conducted using a measurement station equipped with an eight-channel digital thermometer connected to a PC (personal computer), which recorded the measurement data. Type K thermocouples (NiCr-NiAl) with a diameter of 0.85 mm were used for the measurements. The thermocouples were welded (capacitor welding) to the measurement points, which were at the bottom of blind holes drilled in the heat-affected zone to a depth of about 5 mm, i.e., near the fusion line.

## 4. Results and Discussion

### 4.1. Stability of Welding

The Weld Cube Premium v 3.1.55 software from Fronius allows for the recording of welding data, including welding current and voltage values during the process, with a time interval of 0.1 s. Table 8 shows the welding current and voltage graphs for power source No. 1 LEAD and power source No. 2 TRAIL (see also Figure 2b), which were recorded during the preparation of samples with different welding speeds. The analysis of the graphs indicates that the welding process was carried out stably. Only in the case of sample No. 3, for the LEAD welder, can we observe a change in the current and voltage values at the ignition point. This is mainly the effect of using robotic welding and stable welding sources [68]. Automation eliminates errors resulting from manual guidance of the torch, i.e., its tilt, distance from the weld pool, and movement speed (linear welding speed). This ensures that a constant linear welding energy is maintained. This, in turn, is important for eliminating local microstructural changes that can cause structural notches. Parameter stability reduces residual stresses [6] and their accumulation in places with significantly different mechanical properties (hardness, strength).

### 4.2. Macrostructure

**Figure 3:**
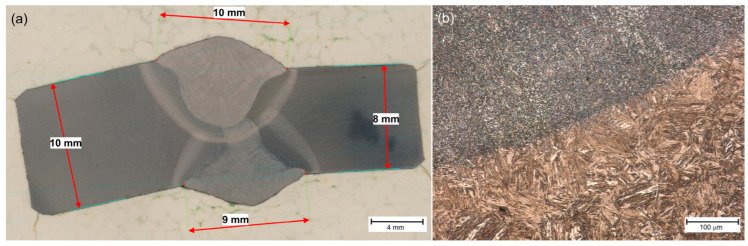
Sample No. 1—(**a**) macro-structure, (**b**) fusion line.

**Figure 4:**
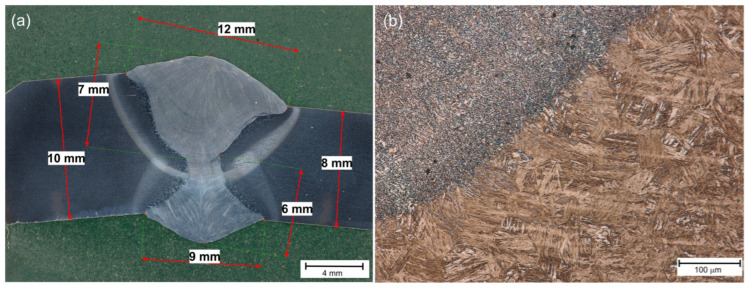
Sample No. 2—(**a**) macro-structure, (**b**) fusion line.

**Figure 5:**
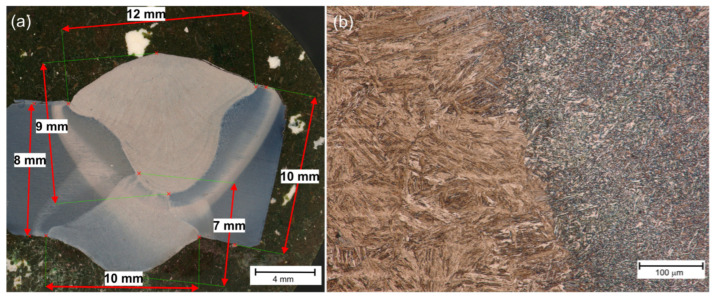
Sample No. 3—(**a**) macro-structure, (**b**) fusion line.

Figure 3, Figure 4 and Figure 5 present the macroscopic images of all three samples. Based on their evaluation, a slight shift in the surface weld relative to the edge weld was observed, mainly in Sample No. 3 (Figure 5). This phenomenon was caused by the positioning of the torch relative to the sample. The effect of Adler’s reagent, typically used to evaluate the macroscopic structure of welded joints [9], showed the correct shape of the welds and a slight overlap (1–2 mm; Figure 4 and Figure 5) of the heat-affected zones of the face and root beads. This means that the welding parameters, i.e., the linear arc energy and the torch angle, were correct. In addition, due to the use of tandem welding (TWIN), the shape of the weld face is narrower at the bottom and wider at the face. The larger energy introduced by the first wire (LEAD) caused a deeper penetration, while the second wire (TRIAL) filled the upper part of the joint. In this way, the weld groove was completely filled in a single run. It also causes the weld structure to be uniform. In the case of multi-run welding, individual runs are visible, and the material is heated more than once, which is not the case in tandem welding [69]. Figure 3a, Figure 4a and Figure 5a show a typical structure of a tandem steel joint. It should be noted, however, that the use of twin-wire welding may lead to an enlargement of the heat-affected zone compared to standard welding [32]. Furthermore, based on a macroscopic study, one can conclude that all welds are free from defects like porosity, inclusions, and cracks [70], etc.

The critical place in welded joints is the so-called fusion line, i.e., the boundary of the weld and the heat-affected zone (HAZ). In these places, so-called luck of fusion can also occur, i.e., there is a lack of partial melting of the welded material [26]. Figure 3b, Figure 4b and Figure 5b show examples of fusion lines. These places are also free from defects for each of the tested joints.

**Figure 6:**
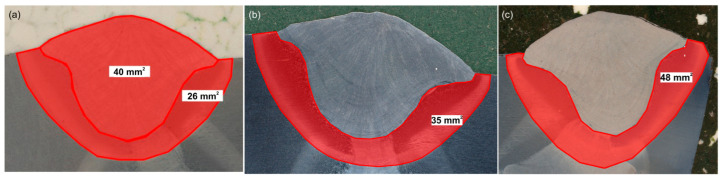
Heat affected zone (HAZ) of samples—(**a**) No. 1, (**b**) No. 2, (**c**) No. 3.

Using optical microscope software, the HAZ surface area of each sample was measured. The results are shown in Figure 6. The measurements indicate that reducing the welding speed resulted in an increase in the HAZ surface area. For Sample No. 1, it was 26 mm^2^, for Sample No. 2, it was 35 mm^2^, and for Sample No. 3, it was 48 mm. A smaller HAZ means a faster cooling rate, which can result in reduced impact strength. In turn, a reduction in the cooling rate (i.e., a larger HAZ) reduces the strength of the material. This relationship does not always hold, as it depends on the actual cooling rates and not only on the HAZ size [8].

### 4.3. Welds Microstructure

Despite the high carbon equivalent CEV value (about 0.4% [9], for example, materials see Table 1), S1100QL and S1300QL steels belong to the group of difficult-to-weld steels, provided that a high level of process technology is maintained. Proper control of the heat input to the welded joint and proper control of the cooling time in the temperature range of 800–500 °C helps minimize the risk of hydrogen cracking [71]. For fine-grained steels with high strength, this time is typically in the range of 5 to 20 s [72].

The microstructure of the S1100QL base material consists of martensite [1]. According to other sources [8], the microstructure of this steel may also contain bainite and small amounts of ferrite. This may occur especially after welding processes, which result in an average cooling rate.

**Figure 7:**
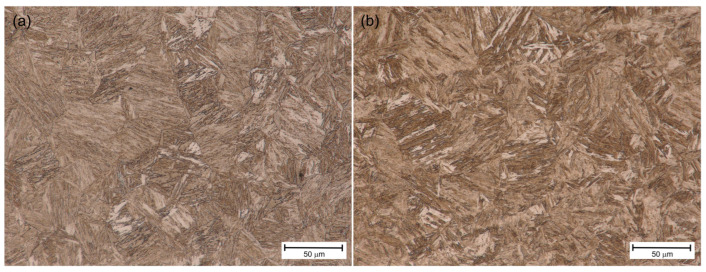
HAZ microstructure of sample No. 1—(**a**) S1100QL, (**b**) S1300QL.

**Figure 8:**
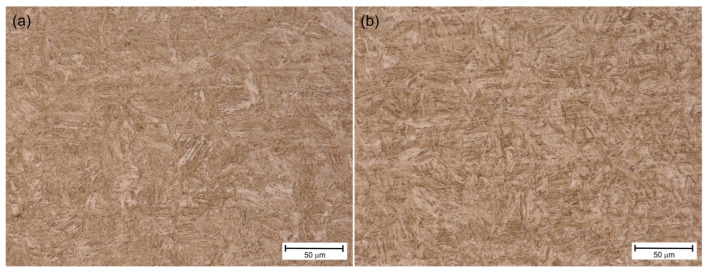
HAZ microstructure of sample No. 2—(**a**) S1100QL, (**b**) S1300QL.

**Figure 9:**
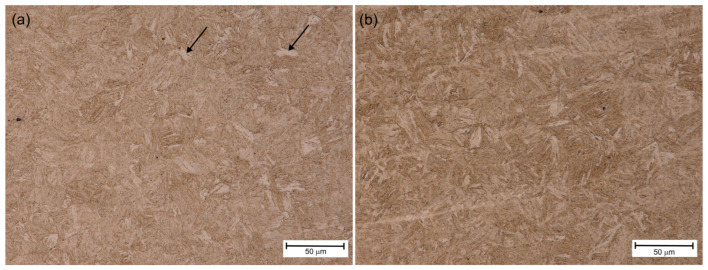
HAZ microstructure of sample No. 3—(**a**) S1100QL; arrows point at bianitic ferrite, (**b**) S1300QL.

Figure 7a, Figure 8a and Figure 9a show the HAZ microstructure of S1100QL steel of the tested joints. In all cases, there is an acicular microstructure typical of this steel. Based on the literature on the subject [1], it can be assumed that it is martensite. In addition, bright areas can be recognized, directed by arrows, e.g., in Figure 9a. This is probably bianitic ferrite. When comparing the microstructures in this respect, it can be seen that for the variant cooled the slowest (sample No. 3—Figure 9a), the needle microstructure is less distinct, and there are slightly more areas that can be identified as bainitic ferrite. This may mean that a tendency towards bainitic transformation is noticeable, especially at a welding speed of 80 cm/min (and the remaining welding parameters adopted—see Table 6).

In turn, Figure 7b, Figure 8b and Figure 9b show the HAZ microstructure of the S1300QL steel of the tested joints. This steel has even greater strength, which means that martensite is present in the microstructure of the steel in the delivery state. As can be seen in the mentioned figures, the welding heat cycle causes the formation of a microstructure that does not significantly differ from the microstructure of the S1100QL steel (with applying the light microscopy analysis method). An acicular structure and bright areas, probably bainitic ferrite, are also visible. Taking into account the chemical composition of the steel and the results of the studies described later in this article, it is also possible that at higher welding speeds (i.e., faster cooling), the microstructure contains martensite and self-tempered martensite.

**Figure 10:**
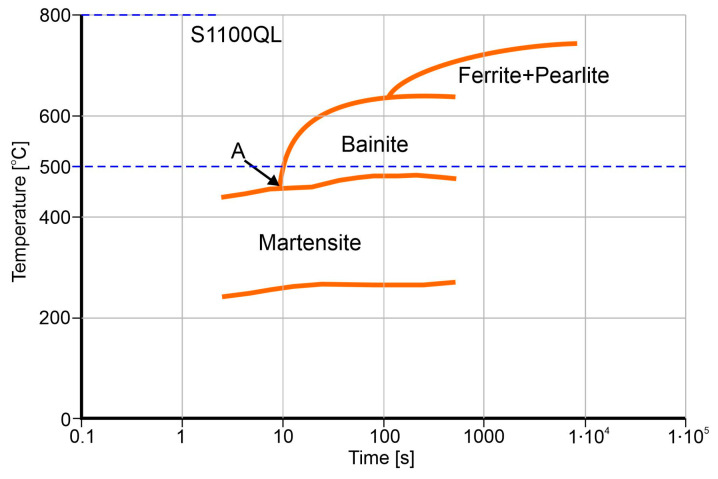
CCT diagram for S1100QL steel; copyright to this figure belongs to the publisher of [8].

In order to understand the microstructure of both steels after welding thermal cycles, it is necessary to analyze the CCT diagrams (Continuous Cooling Transformation). In Figure 10, the CCT diagram for S1100QL steel prepared based on the diagram provided in the literature [8] is presented. As can be seen, cooling rates in the range of 5 to 20 s, which are often encountered in GMAW welding, will cause the formation of a martensitic or martensitic-bainitic structure. For S1300QL steel, the transformation lines in the CCT diagram are shifted to the right side [1]. The point A location (Figure 10) at shorter times suggests that this steel will be more exposed to the formation of a martensitic-bainitic structure during medium thermal cycles. Analysis of the transformations in the studied case based on the difference in the position of the curves in the CCT diagrams is difficult due to the different cooling rates in both steels because the joint is not symmetrical, i.e., the difference in the thickness of the welded elements is 2 mm (see Figure 1).

However, when analyzing the CCT diagram (Figure 10), it is worth noting that the onset of the martensitic (Ms) and bainitic transformations, including point A, occurs at relatively high temperatures. The martensitic transformation begins in the temperature range of approx. 460–490 °C. In turn, the position of point A and the curve for the bainitic transformation indicate that, in the case of typical welding cycles, lower bainite will be formed, characterized by carbides dispersed throughout the volume of bainitic ferrite. This provides mechanical properties, including hardness and strength, comparable to those of tempered martensite. Taking into account the above considerations and the assumption that the cooling curve crosses with the line of the beginning of the bainitic transformation at a level of approx. 500 °C, this may result in the formation of a microstructure consisting of a small amount of bainite (upper) in the martensite matrix. This reasoning is confirmed especially in Figure 9.

### 4.4. Visual Tests—VT

The technological aspect of the experiments and research is also essential. For this reason, research on the quality level should be conducted according to the standards. The results of the study are presented in Table 9. All three joints meet the requirements at the B level of quality.

### 4.5. Mechanical Properties of Welds

#### 4.5.1. Static Tensile Tests

Table 10 presents the results of static tensile tests performed in the direction perpendicular to the welding direction, where S01—cross-sectional area before the test, F—maximum force. The test can be considered untypical because it is necessary to remember the different thicknesses of the welded materials and the profile of the joints (see Figure 1). The samples cracked in the welds. It was expected because each of the welded steels has a higher tensile strength (UTS) than the welding wire used (UTS max. 1080 MPa)—compare Table 3 and Table 5. The significance is that the results obtained excellent levels because the weld strength reached the maximum strength of the welding wire used. The reasons for this may be related to the cooling rate and partial mixing with the welded material, the chemical composition of which favors the formation of needle-like structures. It should be noted that filler metals with a strength of 900 MPa or even 1100 MPa are available for welding this type of steel [6,8]. Probably due to costs, construction manufacturers more often use filler metals with lower yield strengths.

**Figure 11:**
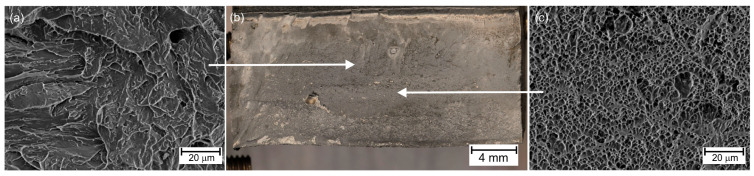
Fracture of tensile test sample No. 1—(**a**) quasi-brittle fracture, (**b**) macroscopic view of fracture, (**c**) ductile fracture.

The samples broken in the static tensile test were observed using scanning electron microscopy (SEM). This research technique also allows for recognizing fracture types on a microscale. Figure 11 shows the observation results of sample No. 1, i.e., for the highest linear welding speed. Quasi-brittle fracture areas (Figure 11a) and ductile fracture areas (Figure 11c) were observed in the fractures. This is an expected result. It must be remembered that the fracture occurred in the weld and, therefore, in a material with a low carbon content (approx. 0.1%wt—see Table 4). If martensite was formed there, then the tetragonality of the crystallographic lattice cell of such martensite is small. This causes a lower state of structural stress. In the case of low-carbon bainite, the precipitation and solution strengthening will play a greater role. It is also the reason for expecting the material to be less brittle.

The strength of the welded joint and the associated fracture mode may also depend on the presence of precipitates around which the plastic flow of the material develops, creating characteristic craters. In most cases, such precipitates are sulfides, but as the authors of the paper discovered [9], they may also be compounds with the participation of Ca and Al, and in the paper [70] authors indicate spherical precipitations contain Mn, O, Ti, S, and Ti.

#### 4.5.2. Hardness Tests

**Figure 12:**
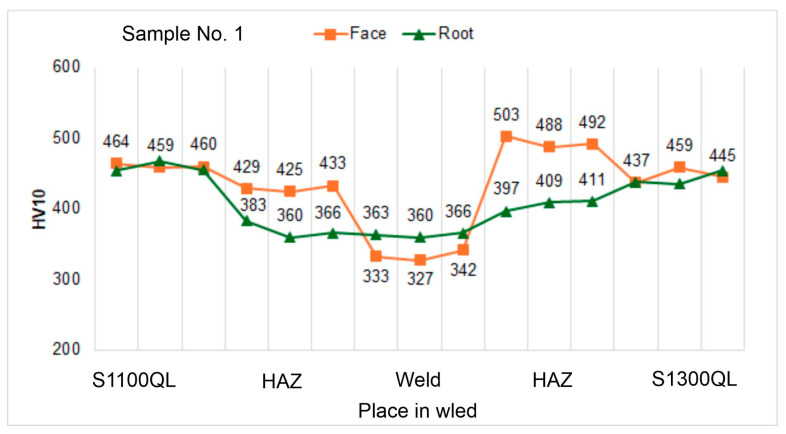
Hardness distribution on the cross-section of sample No. 1.

**Figure 13:**
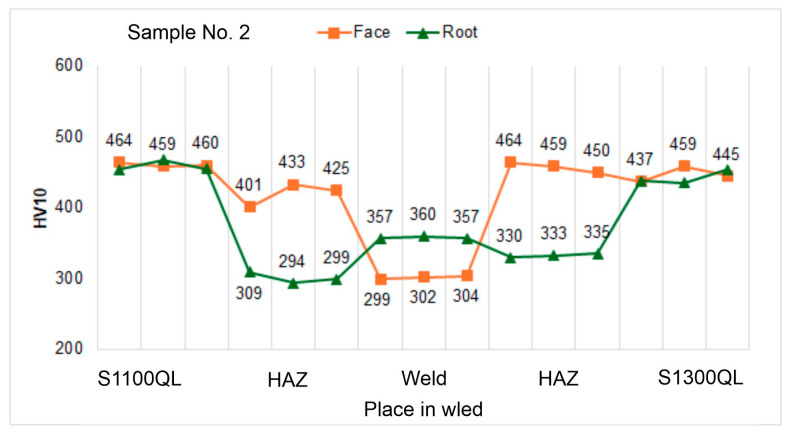
Hardness distribution on the cross-section of sample No. 2.

**Figure 14:**
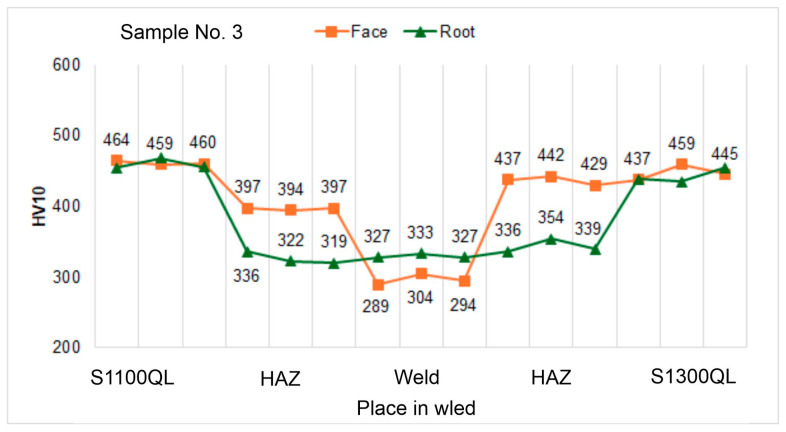
Hardness distribution on the cross-section of sample No. 3.

Analyzing the hardness graphs, presented in Figure 12, Figure 13 and Figure 14, it was observed that the highest values were measured in the HAZ of the S1300QL steel near the face of the welds. Significantly, the hardness for the highest welding speed (100 cm/min; sample No. 1) is about 50 HV higher than the hardness of the parent material. With the decrease in the welding speed, this difference decreases significantly—for sample No. 2 (welding speed 90 cm/min), it is only about 15 HV, and for sample No. 3 (100 cm/min), the hardness in HAZ and the base material is comparable. The last result, i.e., for sample No. 3, is also confirmed in the work [9]. The authors of this work indicate, however, a sharp decrease in hardness at the end of HAZ from the side of the base material by as much as about 100 HV. This phenomenon was not observed for any of the samples, Nos. 1 to 3. The hardness distribution on the root side is different. In this case, the HAZ hardness is much lower than the base material hardness by about 50 to 120 HV, with a smaller decrease noted for sample No. 1, i.e., the highest welding speed.

However, when we analyze the results for the S1100QL steel, we can see that for each sample, the HAZ hardness is lower than the base material and that it decreases with welding speed. For samples No. 1, No. 2, and No. 3, the difference is approximately 30 HV, 60–35 HV, and above 60 HV, respectively. When we analyze the hardness distribution on the root side, it can be observed that the hardness of HAZ is significantly lower than the hardness of the parent material by up to approx. 160 HV—similar to the second steel, although to a lesser extent.

When the hardness of the weld of all samples is considered, it is natural that lower results are noted. This results from the mechanical properties of the filler metal, the strength parameters of which are lower than those of the welded steel.

In general, the hardness profile depends on these factors and the phase transformations occurring in the HAZ. The hardness profile with a decrease in the weld has been observed by the authors of the work [9]. On the other side, in work [70] describing the welding of S960QL steel, the authors reported an increase in the hardness of HAZ and comparable hardness of the weld and the base material.

#### 4.5.3. Impact Test

**Figure 15:**
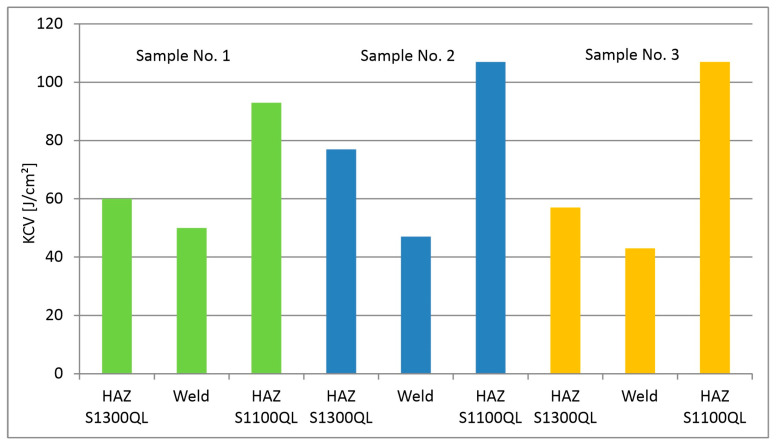
Impact toughness KCV graph for different areas of the welds.

Table 11 and Figure 15 present the results of the impact test conducted for Sample No. 1, Sample No. 2, and Sample No. 3, while the impact toughness (KCV) is calculated as KCV = KV/S0 J/cm^2^, when S0 = 0.6 cm^2^ and KV is impact energy (test value energy), and S0—cross-sectional area under the notch. The test was performed at a temperature of −40 °C.

As one can see in Figure 15, the lowest impact strength in individual joints was found for the weld. In addition, the value decreases with decreasing welding speed, although the differences between samples are not significant. This is unusual because lower welding speed means slower cooling. It is likely that the decrease in impact strength, in this case, is not related to the martensitic matrix but depends more on the state of precipitates, which is indicated by the presence of alloying elements and favorable conditions for diffusion.

Comparing the results for HAZ, it can be seen that the impact strength on the side of the S1300QL steel is clearly lower. It can also be stated that for this steel, the welding speed does not clearly affect the trend of impact strength changes. The situation is different for the S1100QL steel, which has lower impact strength for the highest cooling rate (sample No. 1).

The obtained impact strength values are confirmed in the literature. For steel S1300QL, the authors of the paper [9] obtained impact strength (Charpy V) at −40 °C in the range of 42 to 60 [J/cm^2^]. Whereas for steel S1100QL, the authors of the paper [8] report a fractured work of approx. 86 J, i.e., approx. 106 J/cm^2^ for standard impact test samples.

### 4.6. Thermal Cycles Analysis

The purpose of measuring thermal cycles during the welding process was to determine the actual cooling rate ranges expressed as cooling times in the temperature range of 500 to 800 °C (t8/5 s) occurring at the welding speeds used in the experiments. The use of t8/5 time in welding process studies and the study of thermal cycles by temperature measurements using thermocouples is often used [1,6,12,72].

The tests were conducted many times to obtain due to the significant number of factors that may affect the measurement results. The place where the thermocouple is welded in the HAZ has the strongest effect, i.e., the distance from the weld pool. In the case of thicker materials (so more than 2 mm), the location can be considered in the Cartesian system, where welding is carried out in the direction of the Y axis, and the Z axis is perpendicular to the plane of the joint face. With such an orientation, the position of the thermocouple end will be determined by coordinates in the X-Z plane. This can also be explained differently, i.e., how far the measuring end of the thermocouple is located from the root and from the weld line in the direction perpendicular to it. The method of drilling holes from the root, adopted in the experiments, to locate the thermocouple end closer to the welding heat source, can cause differences in the position of individual thermocouples.

**Figure 16:**
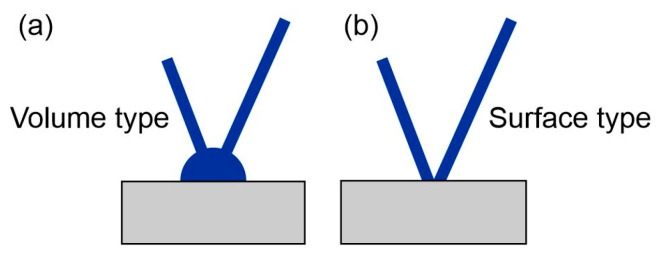
Two types of thermocouple joints for research material—(**a**) volume type, (**b**) surface type [73].

**Figure 17:**
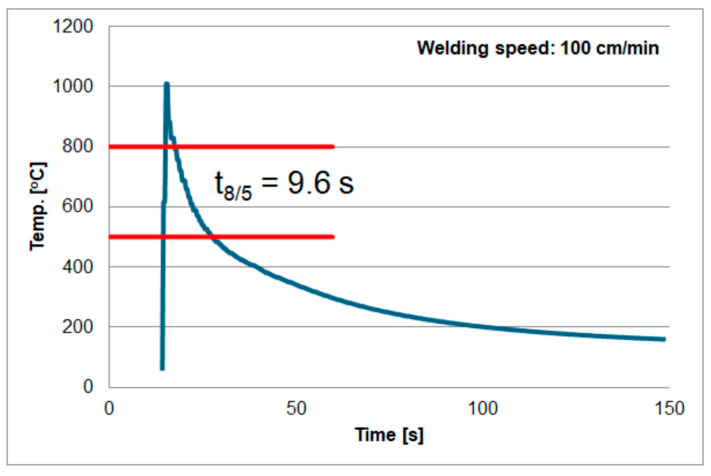
Thermal cycle of welding speed of 100 cm/min.

**Figure 18:**
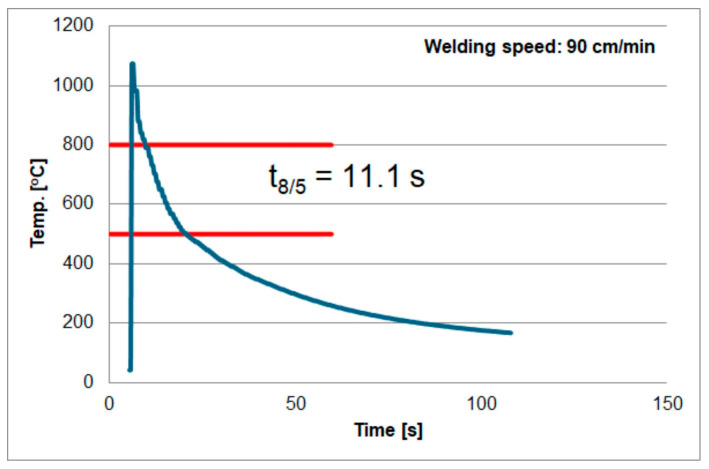
Thermal cycle of welding speed of 90 cm/min.

**Figure 19:**
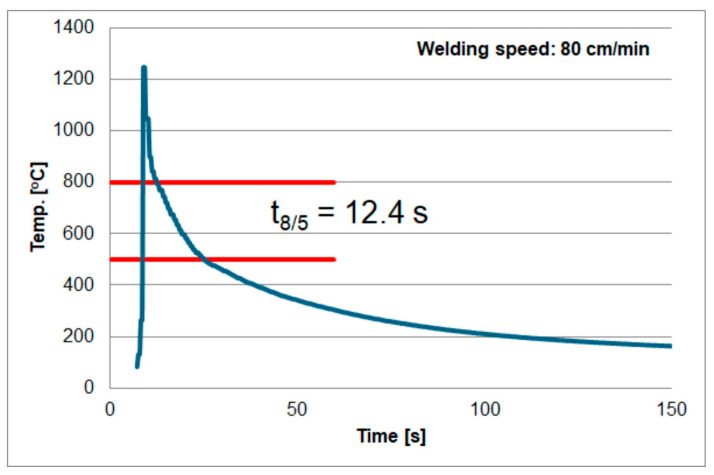
Thermal cycle of welding speed of 80 cm/min.

The second factor that may affect the measurements was described in the paper [73]. According to the authors, the volume of the measuring weld can affect the measurement accuracy. The authors consider two cases assuming a metallurgical connection (i.e., by welding) of the thermocouple to the tested material. They are shown in Figure 16. The method shown in Figure 16a is called the volume type of thermocouple. The surface type is shown in Figure 16b). According to the authors, measurements with the first type of thermocouple (volume) may be less accurate due to the volume of the measuring weld and the surface area of the thermocouple contact with the tested material. As mentioned earlier, in the tests, the thermocouple was mounted at the bottom of blind holes using the capacitor welding method (electric current pulse). This means that it was not possible to determine what type of contact (thermocouple) was created after welding. The above factors could have caused different results for welding under the same conditions. Based on these experiments, the results shown in Figure 17, Figure 18 and Figure 19 were obtained. Temperature measurement was carried out in the HAZ of S1300QL steel. Based on this, the cooling time t8/5 was calculated. For sample number 1, the t8/5 value was the lowest, at 9.6 s, for sample number 2, the t8/5 value was higher, at 11.1 s, and the highest cooling time was obtained for sample number 3, at 12.4 s. The difference between the lowest and highest values was 2.8 s. The difference in welding speed was 20 cm/min.

### 4.7. Thermal Cycle Simulation

The samples for thermal cycle simulation were taken from S1100 steel sheet with a dimension of 100 × 10 × 10 mm^3^ and S1300 steel sheet with dimensions of 100 × 10 × 8 mm^3^. The samples were placed in a thermal cycle simulator, heated to a temperature of about 1000 °C, and cooled with a controlled time of 5 s in the temperature range of 800–500 °C.

The analysis of measurement results is described in Section 4.6. shows that the shortest cooling time t8/5 is 9.6 s. However, the time t8/5 = 5 s was assumed for the thermal cycle simulation. The reason is the assumption that conditions can be stiffer than those recorded. Moreover, the literature analysis [12] indicates that such a time, t8/5, was also taken into account during the tests.

#### 4.7.1. Microstructure of Samples After Simulation

**Figure 20:**
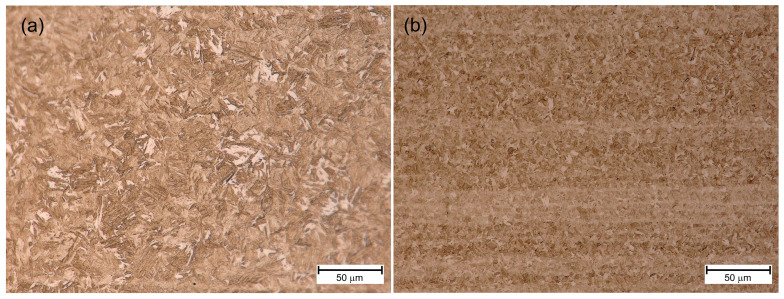
Microstructure of S1100QL—(**a**) base material (**b**) heat treated 1000 °C, t8/5 = 5 s.

**Figure 21:**
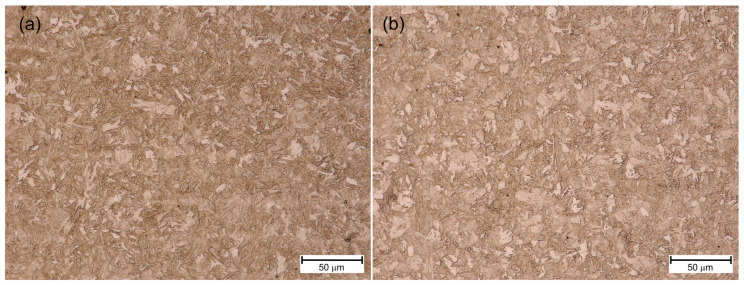
Microstructure of S1300QL—(**a**) base material (**b**) heat treated 1000 °C, t8/5 = 5 s.

Figure 20 and Figure 21 show the microstructure of S1100QL and S1300QL steel in the basic state and after simulated thermal cycles of 1000 °C and cooling time t8/5 = 5 s, imaged by means of a light microscope. The large refinement of the microstructure of S1100QL steel after the thermal cycle is worth noting—Figure 20b.

**Figure 22:**
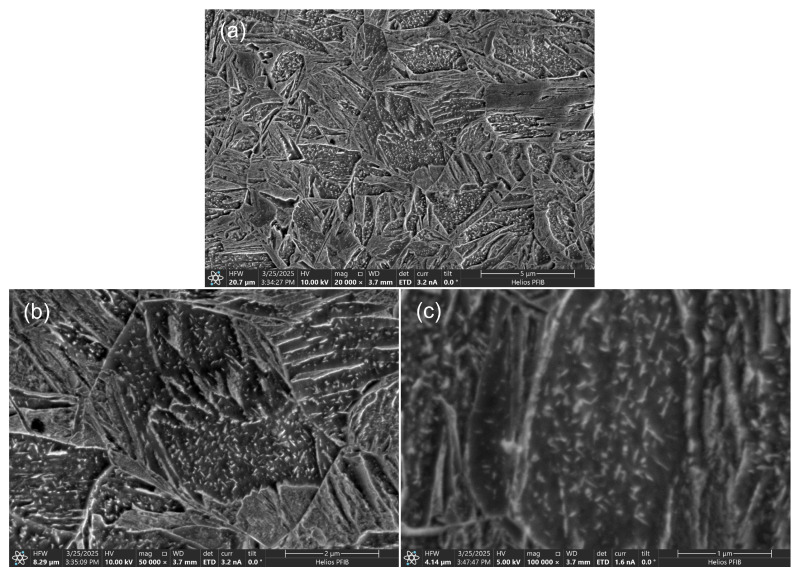
SEM microstructure of S1100QL steel after thermal cycle simulation—(**a**) martensite and lower bainite, (**b**) in central part, microstructure within former austenite grain, (**c**) carbides on the bainitic ferrite laths—lower bainite.

**Figure 23:**
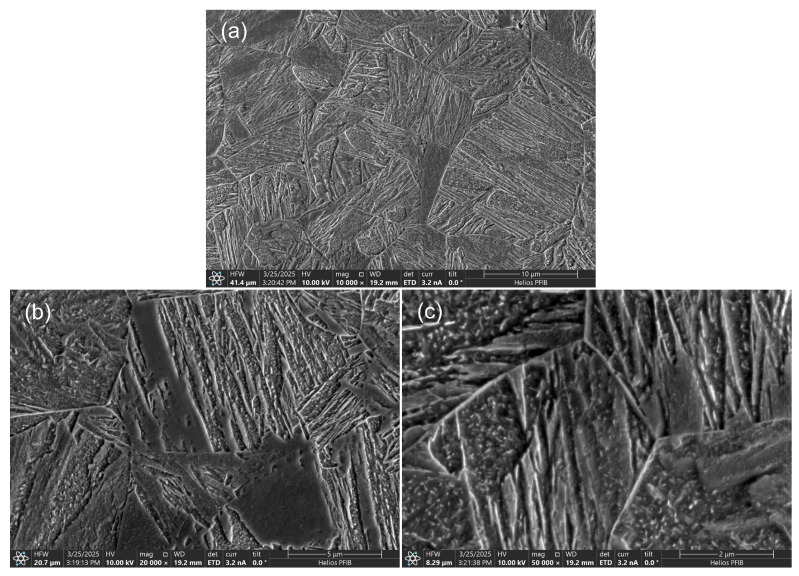
SEM microstructure of S1300QL steel after thermal cycle simulation—(**a**) martensite, (**b**) martensite within the former austenite grains, (**c**) discrete fine transitional iron carbides on martensite laths.

**Figure 24:**
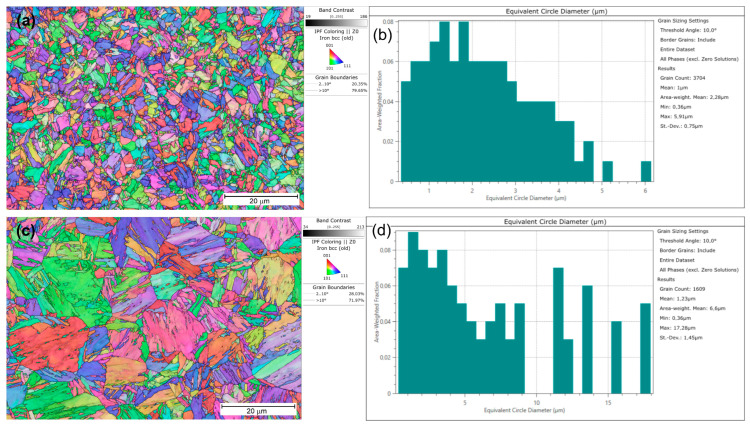
EBSD analysis results. (**a**) IPF for S1100QL steel; (**b**) grain size distribution for S1100QL steel; (**c**) IPF for S1300QL steel; (**d**) grain size distribution for S1300QL steel.

For samples, additional tests were conducted using SEM/EBSD (electron backscatter diffraction) to determine the changes induced by heating to 1000 °C. Figure 22 shows the microstructure for S1100Q steel, which is composed of martensite and lower bainite (carbides are visible on the bainitic ferrite laths, see Figure 22c), with a small amount of upper bainite. Figure 23 presents the microstructure for S1300QL steel, which is martensitic. Discrete fine transitional iron carbides are visible on the martensite laths, most likely as a result of self-tempering, since the Ms temperature (temperature of martensite start) of this steel is approximately 440 °C [1]. Interestingly, the prior austenite grain size, as determined by EBSD (Figure 24) in S1100QL steel, is significantly smaller (2.28 µm) than that in S1300QL steel (6.60 µm). This is most likely due to the higher content of nickel and manganese (austenite-forming elements) in S1300QL steel, which resulted in the formation of austenite at a lower temperature during heating, allowing more time for austenite grain growth.

#### 4.7.2. Mechanical Properties of Samples After Simulation

Mechanical properties were tested in terms of hardness and impact strength. Impact strength was determined at a temperature of −40 °C using samples with dimensions of 55 × 10 × 7.5 mm^3^ and a Charpy-V notch. Impact toughness (KCV) was determined according to the equation KCV = KV/S0. The test results are presented in Table 12.

As can be seen, the impact strength for both steels is significantly higher than 27 J despite very rapid cooling. When comparing these results with the results shown in Table 11, it can be seen that the impact strength of the S1300QL steel is at the same level, and the result for the S1100QL steel is lower by about 20 J. In turn, when comparing the hardness results (Table 12 and Figure 12), a similar level was achieved by the S1100QL steel, while the S1300QL steel shows a higher hardness by 30 HV. However, it should be remembered that the simulation conditions were stiffer (t8/5 = 5 s) than those measured during the welding experiment (t8/5 = 9.6 s for a welding speed of 100 cm/min).

When comparing these results with the results of the studies presented in work [12], in which the authors described the studies of steel S960QL, S1100M, and S1300QL on samples subjected to simulated thermal cycles, including the cooling time t8/5 = 5 s, one can see a significant difference in impact strength. As mentioned in the article, the impact strength was below 27 J. This is a significant difference. This may be the result of the specific steels used in both studies. Comparing the CEV and CET carbon equivalents, one should notice the differences in the disadvantages of the steels described in the article. For the steel S1100M, it is CEV = 0.68% and CET 0.39%, and for the steel S1300QL, it is CEV = 0.956% and CET = 0.56% [12]. The carbon equivalents for the tested steels are given in Table 13, where the CEV equivalent was calculated according to Equation (1) and the CET equivalent according to Equation (2) [74].

(2) $$\mathrm{CET}=C+\frac{\mathrm{Mn}+\mathrm{Mo}}{10}+\frac{\mathrm{Cr}+\mathrm{Cu}}{20}+\frac{\mathrm{Ni}}{40}\%\mathrm{wt}$$

## 5. Conclusions

The main goal of the welding experiment was to examine the impact of changes in welding speed on the properties of the welded joint, performed using the Fronius TWIN tandem welding technology. Based on the conducted tests and analyses, the following conclusions have been drawn:

- Application of a tandem welding allows to welding with stable parameters of the process and with high efficiency. Moreover, conducting process with all the applied parameters enables the achievement of full penetration and metallurgical continuity.
- Reducing the welding speed resulted in an increase in the HAZ surface area and a higher linear energy.
- The microstructure of S1100QL steel contains martensite and bainite. In the case of welding with higher speeds, it will mainly involve lower bainite. On the other hand, the microstructure of S1300QL steel for higher speeds contains martensite and tempered martensite. In the case of lower speeds, it is possible to form bainite.
- S1100QL steel tends to refine the austenite grain and, consequently, refine the martensitic structure under the influence of the rapid thermal cycle of welding t8/5 = 5 s. Acceptable joint strength was obtained. UTS reached maximum values for the applied welding filler metal.
- The tested steels can achieve impact strength levels significantly above 27 J at −40 °C even for a rapid cooling rate (i.e., time t8/5 = 5 s).
- Reducing the welding speed by 20% prolonged the t8/5 cooling time, which led to a decrease in hardness levels in both the weld and HAZ areas on both sides of the materials, as well as an increase in the fracture work value at −40 °C.
- In the analyzed case, the optimal welding speed was 80 cm/min.

## Figures and Tables

**Table 1:** Chemical composition of S1100QL steel—the melt analysis, %wt.

Steel	C	Si	Mn	P	S	Cr	Ni	Mo	CEV
S1100QL	0.16%	0.20%	1.25%	0.011%	0.001%	0.20%	0.70%	0.60%	0.54%
	V	Ti	Cu	Al	Zr	Nb	B	N	
	0.04%	0.002%	0.01%	0.06%	0.002%	0.01%	0.001%	0.003%	

**Table 2:** Chemical composition of S1300QL steel—the melt analysis, %wt.

Steel	C	Si	Mn	P	S	Cr	Ni	Mo	CEV
S1300QL	0.23%	0.09%	0.95%	0.008%	0.002%	0.36%	1.21%	0.46%	0.64%
	V	Ti	Cu	Al	Zr	Nb	B	N	
	0.03%	0.001%	0.01%	0.05%	-	0.01%	-	0.003%	

**Table 3:** Mechanical properties of S1100QL and S1300QL steels.

Steel	YP [MPa]	UTS [MPa]	Elongation [%]	KV [J]	HV10
				(−40 °C)	
S1100QL	≥1100	1250–1450	≥10	≥27	<430
S1300QL	≥1300	1400–1700	≥8	≥27	<450

**Table 4:** Chemical composition of the filler material %wt.

Filler Material	C	Si	Mn	Cr	Ni	Mo
G 79	0.09%	0.70%	1.70%	0.30%	1.85%	0.60%

**Table 5:** Mechanical properties of the filler material.

Filler Material	YP [MPa]	UTS [MPa]	Elongation [%]	KV [J] (20 °C)	KV [J] (−50 °C)
G 79	≥790	≥880–1080	≥16	≥90	≥47

**Table 6:** Welding parameters applied in the experiments.

Parameters	Root Layer	Final Layer LEAD	Final Layer TRAIL
Welding method	MAG	MAG	MAG
Process	Puls	TWIN PMC PSC	TWIN PMC UNIVERSAL
Synergic line (number)	-	4018	3940
Power source	TPS 5000	TPS 600/i	TPS 600/i
Filler material/diameter [mm]	G 79/1.0	G 79/1.0	G 79/1.0
Shielding gas	M21	M21	M21
Gas flow [L/min]	12	15	15
Wire consumption [m]	-	15.27	9.99
Wire speed [m/min]	8.5	19	14
Current [A]	230	300	196
Voltage [V]	22	23.6	22.2
Arc correction	−3	−1.5	−2
Pulse correction	3	0	−2
Welding speed [cm/min]	45	100/90/80	100/90/80
Linear Energy [kJ/mm]	0.54	0.72/0.8/1.1	

**Table 7:** Sample designation.

Sample Designation	Welding Speed [cm/min]
No. 1	100
No. 2	90
No. 3	80

**Table 8:** Welding voltage and current graphs for welding processes.

LEAD	TRAIL
Sample No. 1 (100 cm/min)	
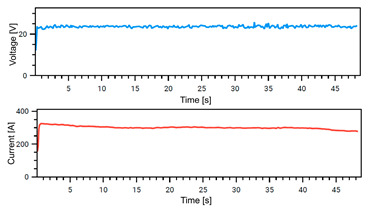	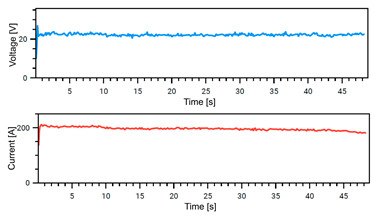
Sample No. 2 (90 cm/min)	
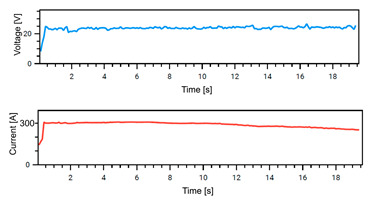	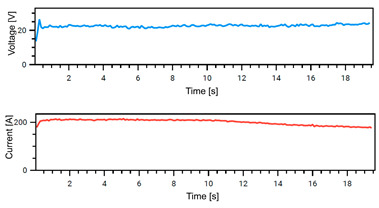
Sample No. 3 (80 cm/min)	
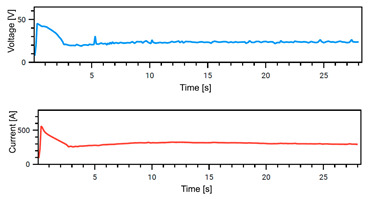	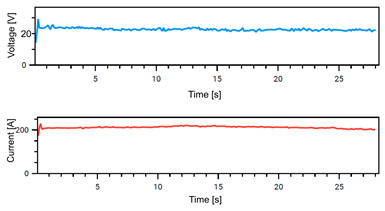

**Table 9:** Visual tests (VT)—result.

Sample	Tested Section [mm]	Welding Discrepancies	Level (Standard [59])		Result
			Required Quality	Achieved Quality	
No. 1	300	No discrepancies	B	B	positive
No. 2	300	No discrepancies	B	B	positive
No. 3	300	No discrepancies	B	B	positive

**Table 10:** Tensile test—results.

Sample No.	Thickness [mm]	Cross-Section S01 [mm^2^]	F [kN]	UTS [MPa]	Fracture Location of the Sample	Result
No. 1	8/10	216	240	1112	In the weld	positive
No. 2	8/10	216	221	1023	In the weld	positive
No. 3	8/10	216	230	1064	In the weld	positive

**Table 11:** Impact test results.

Sample	No. 1			No. 2			No. 3		
Place	HAZ S1300QL	Weld	HAZ S1100QL	HAZ S1300QL	Weld	HAZ S1100QL	HAZ S1300QL	Weld	HAZ S1100QL
KV [J]	36	30	56	46	28	64	34	26	64
KCV [J/cm^2^]	60	50	93	77	47	107	57	43	107

**Table 12:** KCV values for the simulated samples—cooling time of 5 s.

Steel	Post-Thermal Cycle 1000 °C, t8/5 = 5 s			
	KV [J]	S0 [cm^2^]	KCV [J/cm^2^]	HV10
S1100QL	47	0.6	78	459
S1300QL	41	0.6	68	525

**Table 13:** Carbon equivalent for studied steels (see Table 1 and Table 2).

Steel	*CEV* [%]	*CET* [%]
S1100QL	0.58	0.37
S1300QL	0.64	0.42

## Data Availability

The original contributions presented in this study are included in the article. Further inquiries can be directed to the corresponding author.
